# A Cautionary Note: Botulinum Toxin for Cervical Dystonia Reconstituted in Sterile Water: More Pain, Still Gain

**DOI:** 10.1002/mdc3.13356

**Published:** 2021-10-20

**Authors:** Bart E.K.S. Swinnen, Stephanie C. Golsteijn, Anne‐Fleur van Rootselaar, Johannes H.T.M. Koelman

**Affiliations:** ^1^ Department of Neurology and Clinical Neurophysiology Amsterdam University Medical Centers, Amsterdam Neuroscience, University of Amsterdam Amsterdam The Netherlands

**Keywords:** botulinum toxin, injection site pain, sterile water

Intramuscular injection with botulinum toxin is an evidence‐based treatment for cervical dystonia. The injection is evidently accompanied by a certain degree of local injection site pain, but is generally well tolerated.

We report four cervical dystonia cases who were inadvertently injected with botulinum toxin type A (Dysport®) reconstituted in sterile water instead of isotonic saline. All patients reported intense injection site pain at the moment of injection, perceived as much more intense than previous injections (Table 1). No other symptoms were reported. The pain improved rapidly over a couple of minutes but persisted to a lesser degree for 2 hours to 2 days. Effectivity of the botulinum toxin treatment was comparable to previous injections in all patients regarding effect latency, effect duration and symptom improvement as measured on a visual analogue scale.

**TABLE 1:** Characteristics of patients injected with botulinum toxin (Dysport®) reconstituted in sterile water

	Subject 1	Subject 2	Subject 3	Subject 4
Sex	M	M	F	F
Age (yr)	73	44	59	67
Botulinum treatment				
Duration (yr)	21	19	5	12
Dose (IU)	460	260	120	540
Tsui score				
Saline*	10.8	2.8	6	10.3
Sterile water	12	4	7	12
Effect latency				
Saline* (w)	1.5	1.4	1.6	1.5
Sterile water (w)	2	1.5	1	*1 day*
Effect duration				
Saline* (w)	8.7	8.2	7.6	7.3
Sterile water (w)	7	7.5	8	8
Dystonia improvement (VAS)				
Saline*	3.1	1.1	1	3.5
Sterile water	3	3	3	6
Injection site pain				
Intensity	Moderate	Moderate	Moderate	Severe†
Duration	2 days	1 day	2 hr	1 day

Following parameters are described for four patients; sex, age, duration of treatment with botulinum toxin, current botulinum toxin dose, Tsui score^5^ at time of next treatment (ie, approximately 3 months after injection), latency to onset of effect on dystonia, duration of effect on dystonia, extent of dystonia improvement (the latter three parameters were obtained retrospectively at the next injection session approximately 3 months later), intensity and duration of injection site pain.

Abbreviations: yr, years; IU, international units; w, weeks; VAS, visual analogue scale; S, saline; W, sterile water; *, mean of previous 10 injections; †, leading to presyncope.

These cases highlight the importance of proper reconstitution of botulinum toxin (ie, in preservative‐free sterile saline 0.9%), as dissolution in sterile water generates intense injection site pain. To our knowledge, this finding has not as such been reported before. However, it is in line with a recent proposed model to investigate pain, that is, intramuscular injection of sterile water or hypertonic saline.^1^ Clinicians are probably unaware of this association, as a survey amongst dermatological surgeons revealed 3.5% of them to reconstitute botulinum toxin in sterile water.^2^ Next to safeguarding proper reconstitution, several other measures have been reported to decrease injection site pain including precooling with ice, topical lidocaine administration, injection needles with smaller diameter, as well as dilution in lidocaine, preservative‐containing saline or pH‐normalized solutions.

Mechanistically, the injection of a hypotonic fluid generates a sudden osmotic gradient in the muscle tissue leading to a seriously disturbed cellular osmotic homeostasis and hence pain. Botulinum toxin itself is probably not involved in additional pain generation, as injection of pure saline is as painful as botulinum toxin type A dissolved in saline.^3^

Despite the adverse effect of increased injection site pain, these cases indicate that the clinical effectivity of botulinum toxin is probably not affected by dissolution in sterile water instead of saline. As sodium chloride has been reported to facilitate dissociation of free neurotoxin, a certain impact on the pharmacological potency of botulinum toxin can however not be excluded.^4^ Yet, this potential impact does not seem to be of clinical importance. This finding might be helpful in counseling future patients in the event of improper reconstitution.

To conclude, cervical dystonia treatment with intramuscular injection of botulinum toxin reconstituted in sterile water instead of saline is probably equally effective but is associated with transient intense injection site pain.

## Author Roles

(1) Patient care; (2) Manuscript preparation: A. Writing of the first draft, B. Review and critique.

B.E.K.S.S.: 1, 2A

S.C.C.: 1, 2B

A.F.R.: 1, 2B

J.H.T.M.K.: 1, 2B

## Disclosures

### Ethical Compliance Statement

Written informed consent has been obtained from all patients. We confirm that we have read the Journal's position on issues involved in ethical publication and affirm that this work is consistent with those guidelines. Ethical standards as laid down in the 1964 Declaration of Helsinki Ethical guidelines have been followed.

### Funding Sources and Conflicts of Interest

Dr Koelman reports grants from Ipsen, Allergan, Merz. The other authors declare no conflict of interest.

### Financial Disclosures for the Previous 12 Months

The authors declare that there are no additional disclosures to report.
